# From Tweets to Streets: Observational Study on the Association Between Twitter Sentiment and Anti-Asian Hate Crimes in New York City from 2019 to 2022

**DOI:** 10.2196/53050

**Published:** 2024-09-09

**Authors:** Hanxue Wei, Yulin Hswen, Junaid S Merchant, Laura B Drew, Quynh C Nguyen, Xiaohe Yue, Heran Mane, Thu T Nguyen

**Affiliations:** 1 Department of City and Regional Planning Cornell University Ithaca, NY United States; 2 Department of Epidemiology and Biostatistics Bakar Computational Health Sciences Institute University of California San Francisco San Francisco, CA United States; 3 Department of Epidemiology & Biostatistics University of Maryland Maryland, MD United States

**Keywords:** anti-Asian, hate crime, Twitter, racism, social media, machine learning, sentiment analysis

## Abstract

**Background:**

Anti-Asian hate crimes escalated during the COVID-19 pandemic; however, limited research has explored the association between social media sentiment and hate crimes toward Asian communities.

**Objective:**

This study aims to investigate the relationship between Twitter (rebranded as X) sentiment data and the occurrence of anti-Asian hate crimes in New York City from 2019 to 2022, a period encompassing both before and during COVID-19 pandemic conditions.

**Methods:**

We used a hate crime dataset from the New York City Police Department. This dataset included detailed information on the occurrence of anti-Asian hate crimes at the police precinct level from 2019 to 2022. We used Twitter’s application programming interface for Academic Research to collect a random 1% sample of publicly available Twitter data in New York State, including New York City, that included 1 or more of the selected Asian-related keywords and applied support vector machine to classify sentiment. We measured sentiment toward the Asian community using the rates of negative and positive sentiment expressed in tweets at the monthly level (N=48). We used negative binomial models to explore the associations between sentiment levels and the number of anti-Asian hate crimes in the same month. We further adjusted our models for confounders such as the unemployment rate and the emergence of the COVID-19 pandemic. As sensitivity analyses, we used distributed lag models to capture 1- to 2-month lag times.

**Results:**

A point increase of 1% in negative sentiment rate toward the Asian community in the same month was associated with a 24% increase (incidence rate ratio [IRR] 1.24; 95% CI 1.07-1.44; *P*=.005) in the number of anti-Asian hate crimes. The association was slightly attenuated after adjusting for unemployment and COVID-19 emergence (ie, after March 2020; *P*=.008). The positive sentiment toward Asian tweets with a 0-month lag was associated with a 12% decrease (IRR 0.88; 95% CI 0.79-0.97; *P*=.002) in expected anti-Asian hate crimes in the same month, but the relationship was no longer significant after adjusting for the unemployment rate and the emergence of COVID-19 pandemic (*P*=.11).

**Conclusions:**

A higher negative sentiment level was associated with more hate crimes specifically targeting the Asian community in the same month. The findings highlight the importance of monitoring public sentiment to predict and potentially mitigate hate crimes against Asian individuals.

## Introduction

In 2020, as COVID-19 infection began spreading globally, the terms “Chinese virus” and “Kung Flu” were frequently used in public discourse and media coverage [1,2]. This terminology coincided with a rise in discrimination and xenophobia toward Asian and Pacific Islander individuals residing in the United States and a subsequent increase in hate incidents that targeted Asian and Pacific Islander individuals [3]. The New York Times reported nationwide anti-Asian sentiment and violence, affecting individuals of all ages, socioeconomic status, and geographical regions [4]. According to a report by Stop AAPI Hate [5], most anti-Asian hate incidents in the United States occurred in public spaces (68%) and businesses (22%), while 10% of incidents took place online. California and New York were the top 2 states with the most reported incidents [5]. In response to the increasing violence toward Asian individuals, the COVID-19 Hate Crimes Act was instituted in 2021, providing supplementary resources for law enforcement and funding for public education [6]. Several cities, including New York City, launched local initiatives such as the “Stop Asian Hate” campaigns to address the issue at the grassroots level [7].

The effects of discrimination and violence against Asian individuals, which escalated during the COVID-19 pandemic, can be extensive and long-lasting, influencing areas such as psychological and physical health, community belonging, cultural identity, and economic security. Experiences of racial discrimination, which are a notable type of psychosocial stressor, can escalate health risks and worsen health status [8]. For Asian communities, there is a significant correlation between racial discrimination and the prevalence of anxiety, depression, suicidal thoughts, and overall psychological distress [9]. Research on racialized disasters suggests that racial discrimination related to the COVID-19 pandemic may have a detrimental impact on the health of Asian Americans and other minority groups [10,11]. A recent survey study found increasing racial discrimination during the COVID-19 pandemic was correlated with worse self-reported mental and physical health among Asian individuals in the United States [12]. In addition to direct potential health effects, discrimination and xenophobia may lead to economic consequences. In 2020, Asian restaurants experienced nearly 20% less traffic than non-Asian restaurants, translating to an estimated loss of US $7.4 billion in revenue [1].

The large-scale public interactions on social media platforms provide an opportunity to track and measure changes in public opinions and sentiment [13], including discrimination and xenophobia toward minority groups [14]. In addition, social media data can capture population-level attitudes while overcoming the limitations of traditional survey methods, including social desirability bias [15]. Social media activities and real-world hate crimes may be interconnected. Social media platforms can be used to facilitate the dissemination of extreme viewpoints, potentially contributing to the propagation of violent crimes [16]. Social media platforms also reflect public sentiment toward real-world issues and events in real time. Using the data collected from social media platforms, the link between social media sentiment and hate crimes against minority individuals has been investigated in numerous studies [17,18]. Previous work has also demonstrated an increase in solidarity toward minority groups on social media data after hate crimes, notably following the Atlanta spa shootings in 2021 [19].

While anti-Asian hate crimes have been increasing in the United States, there has been limited quantitative research linking social media sentiment to anti-Asian hate crimes. Existing studies on anti-Asian sentiment have primarily explored descriptive aspects of social or news media sources and have illustrated a rising trend during the COVID-19 pandemic [2,18,20]. Yet how online sentiment against the Asian community on social media may be an early proxy or contributor to anti-Asian hate crimes has not been studied. Understanding the link between social media sentiment and real-world hate crimes, particularly hate crimes against Asian individuals and other vulnerable communities, is crucial for creating targeted policy and social interventions. In response to this research gap, our study used quantitative methods to explore the association between anti-Asian Twitter (rebranded as X) sentiment and anti-Asian hate crimes in New York from 2019 to 2022. Our study is motivated by 1 primary research question, that is, is Twitter-derived sentiment toward the Asian community associated with the anti-Asian hate crime rate?

## Methods

### Overview

This research used several data sources, including New York City hate crime data from 2019 to 2022; a total of 868,012 tweets sent from New York State between 2019 and 2022; New York City demographic data in 2020; and New York City unemployment data from 2019 to 2022.

Hate crime data were obtained from the New York City Open Data Portal [21]. Crimes categorized as “anti-Asian” in terms of bias motive were selected for analysis, and we aggregated the counts of anti-Asian hate crimes in New York City at the monthly level from January 1, 2019, to December 31, 2022. Hate crimes are substantially underreported, and, in particular, Asian survivors are less likely to report hate crimes perpetrated against them compared with other survivors [22]. Thus, the actual number of monthly hate crimes was likely an underestimation than what was reported. Furthermore, finer spatial-level visualizations of yearly anti-Asian hate crimes, along with the distribution of Asian communities, were conducted to illustrate the spatial patterns at the police precinct level in New York City. We linked hate crime data with the police precinct boundary data from the New York City Open Data Portal [23]; the police precinct–level Asian population data were calculated by aggregating block-level population data from the 2020 Decennial Census [24]. Based on the existing datasets, we used QGIS and Python via Jupyter Notebook to create all the figures. For Twitter sentiment data, we used Twitter’s application programming interface (API) for Academic Research to collect a random 1% sample of publicly available tweets between January 1, 2019, and December 31, 2022. Our collection was restricted to tweets in English in New York State, including New York City, that included 1 or more of the 10 Asian-related keywords: “Asian,” “Asians,” “Filipino,” “Japanese,” “Korean,” “Nepal,” “Pacific Islander,” “Thai,” “Vietnamese,” and “Chinese.” The word list originated from previous research [25,26]. In cases where tweets lacked city or state data, users’ locations were used instead. If no city or state information was obtainable, we categorized them as having missing values. We applied a support vector machine, a supervised machine learning algorithm, to classify sentiment. The training dataset includes a total of 19,178 prelabeled tweets, consisting of both public sources from Sentiment140 (n=498), Kaggle (n=7086), and Sanders (n=5113) and data manually labeled by our study team (n=6,481). We created distinct binary classifiers for negative and positive sentiments, respectively. The support vector machine has an accuracy of 0.91, an *F*_1_-score of 0.84, and a precision of 0.89 in the categorization of negative sentiment.

Negative binomial models were used to investigate the relationship between hate crimes and Twitter sentiment. Negative binomial models were better for our count data as compared with the Poisson regression given the overdispersed distribution of the monthly counts of hate crimes. In our study, the dependent variable in the negative binomial model was the monthly count of anti-Asian hate crimes in New York City. We first used negative binomial models to investigate the association between the proportion of tweets referencing the Asian community that expressed negative (or positive) sentiment and the counts of anti-Asian hate crimes in New York City in the same month from 2019 to 2022. In addition to the crude models, we ran adjusted models with an indicator variable for the COVID-19 pandemic (before and after the start date of March 2020) and the monthly unemployment rate in New York City in the same month. March 2020 was when the World Health Organization (WHO) characterized COVID-19 infection as a pandemic and when COVID-19 infection began to be referred to as the “China virus”. For our COVID-19 pandemic variable, we used a 0 value for periods before March 2020 and a 1 value for periods from March 2020 onward. This allowed us to determine the social shift that the term “China virus” had on Twitter sentiment and anti-Asian hate crimes. In addition, the unemployment rate is considered a confounder in our study: unemployment could affect psychological well-being [27], which may be reflected in increased frustration and negativity expressed online. A previous study also shows that sentiments of surprise and fear were associated with poverty and unemployment rates since the start of the COVID-19 pandemic [28]. Unemployment may also be linked to the rate of offline hate crimes. Research has shown that periods of high unemployment often coincide with an increase in hate crimes [29,30], as economic distress may exacerbate social tensions and prejudices. Thus, we adjusted for New York City’s monthly unemployment rate based on data from the Bureau of Labor Statistics [31]. In addition, it may take time for sentiment expressed online to be reflected in real-world hate crimes—for instance, a previous study connecting online sentiment with daily worry among Hispanic individuals found a significant 1-week lag [12]. In our main analysis, using the same month data would include a time lag within days and up to 4 weeks. We also used distributed lag models as sensitivity analysis, adding in a 1- and 2-month lag in addition to a 0-month lag time to capture longer time lags [32].

### Ethical Considerations

All data used in this study were publicly available and did not include any personal identifiers. The Twitter sentiment data were analyzed in aggregate, and individual tweets were not identified in any way. The publicly available hate crime data provided by the New York City Police Department were anonymized and deidentified before our analysis, ensuring the privacy of the individuals involved in these incidents. This study was determined exempt by the University of Maryland College Park Institutional Review Board (1797788-1).

## Results

**Figure 1:**
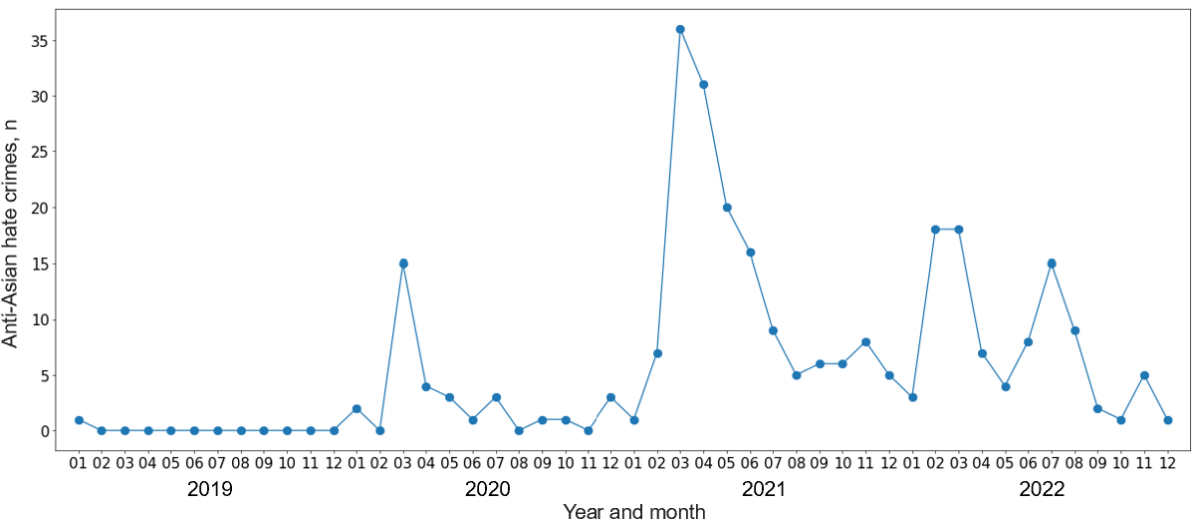
Monthly count of anti-Asian hate crimes in New York City from 2019 to 2022. Data source: New York City Police Department.

Figure 1 [21] illustrates the number of anti-Asian hate crimes in New York City by month. A noticeable increase can be seen starting from March 2020. The peak occurred in March 2021, and a substantial number of hate crimes continued to be reported. In total, there were 1, 33, 150, and 91 hate crimes reported in 2019, 2020, 2021, and 2022, respectively, indicating a dramatic increase during the early stages of the pandemic [21].

**Figure 2:**
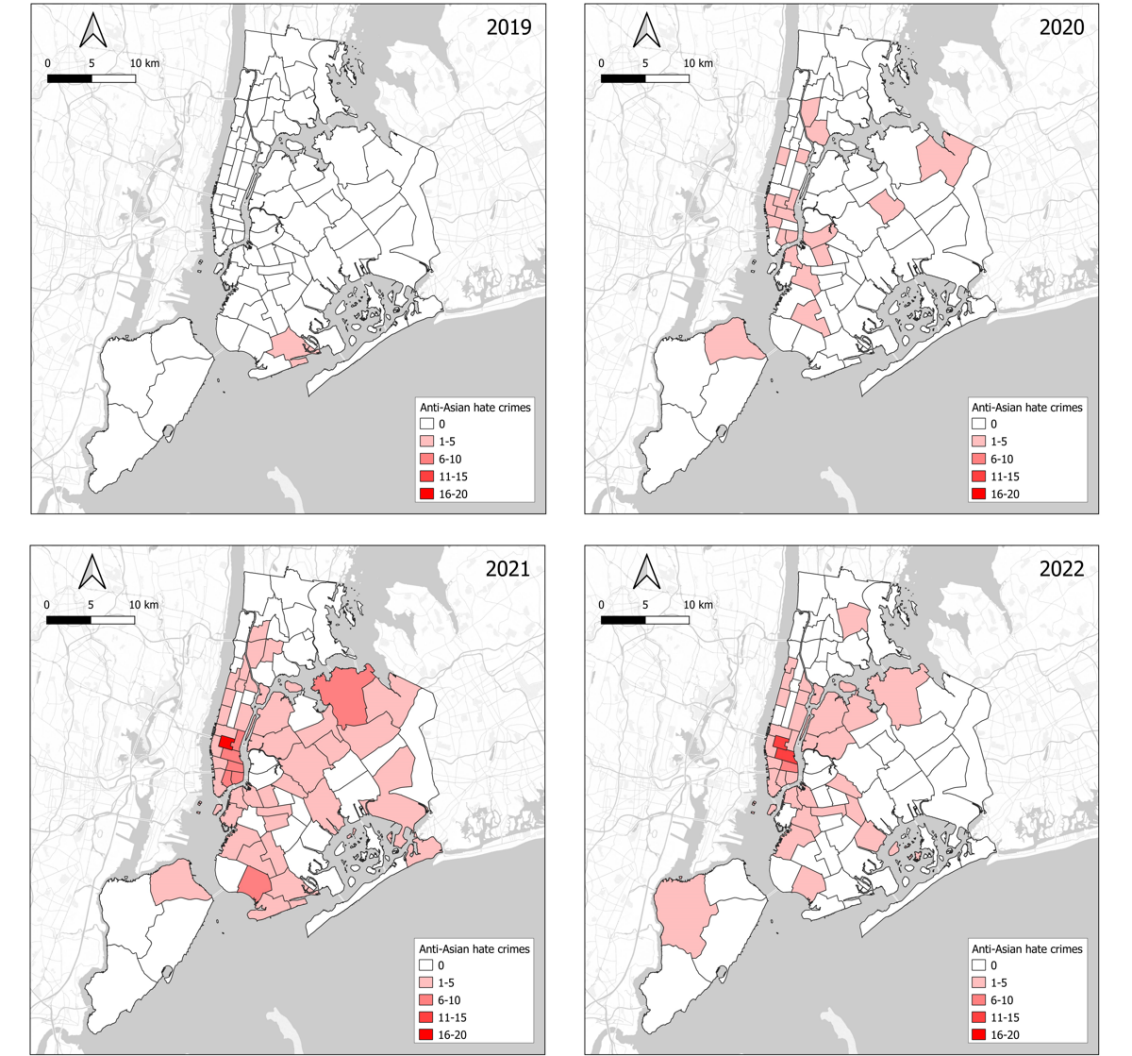
Number of anti-Asian hate crimes by police precincts in New York City from 2019 to 2022. Data source: New York City Police Department and NYC Open Data.

**Figure 3:**
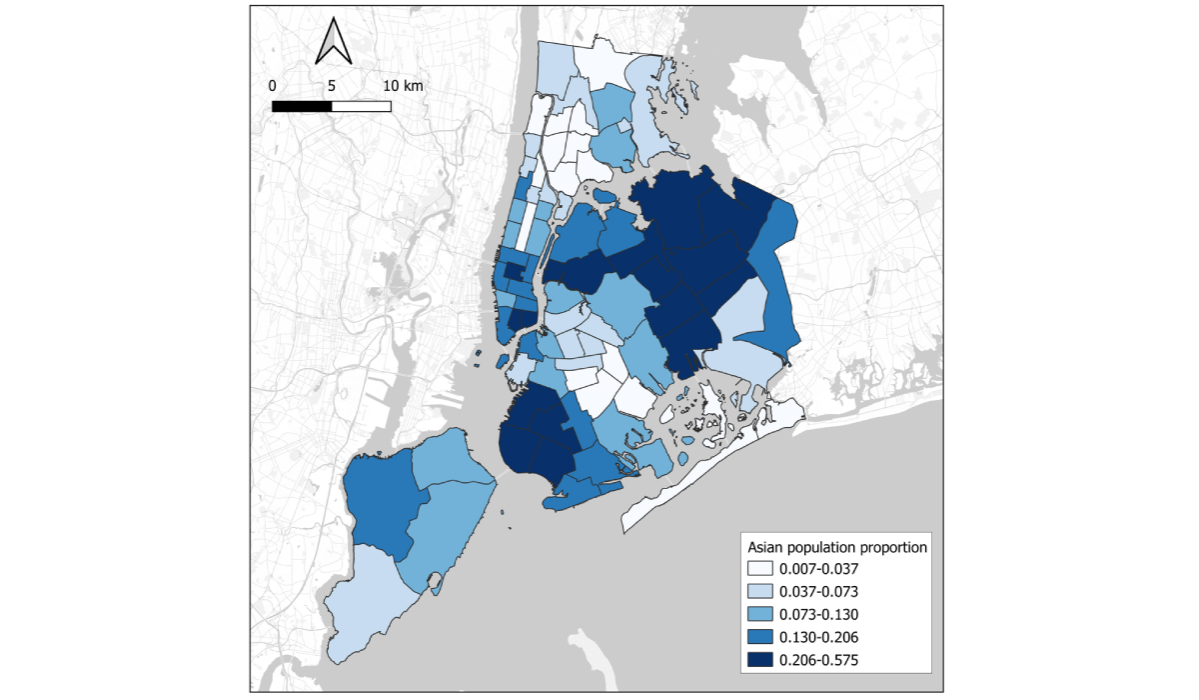
Asian population proportion out of the total population by police precinct in New York City based on 2020 Decennial Census. Data source: NYC Open Data and 2020 Decennial Census.

Figures 2 and 3 [21,23,24] reveal a possible association between anti-Asian hate crimes and the locations of high Asian population densities at the police precinct level. Figure 2 provides an annual overview of anti-Asian hate crime distribution across New York City’s police precincts. It is noteworthy that the frequency of such crimes experienced a surge since 2020, particularly in the Garment District and Union Square regions where there are significant Asian populations. Furthermore, by 2021, an escalation of anti-Asian hate crimes was observed in the northern region of Queens and the southern part of Brooklyn [21,23]. Figure 3 represents the proportion of the Asian population to the total population at the police precinct level in 2020 (for calculation, refer Multimedia Appendix 1). As illustrated in the figure, areas witnessing higher frequencies of anti-Asian hate crimes aligned with regions housing a significant Asian demographic to some extent. At the precinct level, the Kendall tau correlation was 0.28 and the Pearson correlation coefficient (*r*) was 0.33, suggesting that there was a moderate positive relationship between the concentration of Asian populations and the total number of anti-Asian hate crimes [23,24].

**Figure 4:**
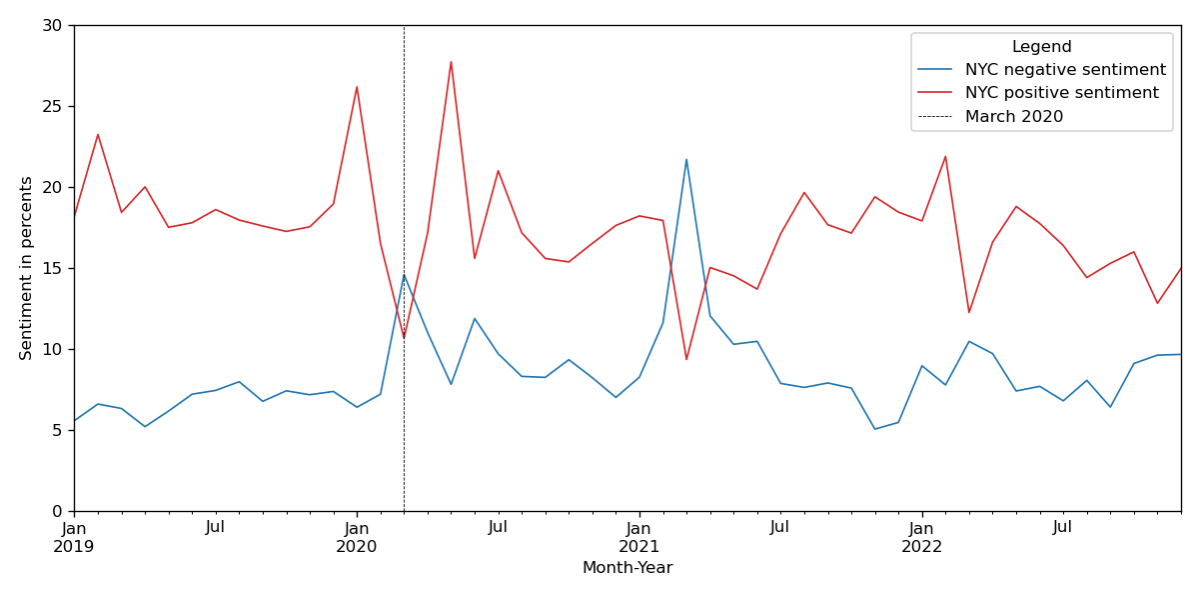
Negative and positive rates of Twitter sentiment toward the Asian community in New York City (NYC) by month from 2019 to 2022. The vertical line in March 2020 marks the point when the COVID-19 pandemic began to spread nationally in the United States.

Figure 4 illustrates the temporal fluctuations of positive and negative Twitter sentiments for tweets containing the term “Asian” for New York City on a monthly basis. Despite the observable fluctuations in sentiment trends, there was an overall downtrend in positive sentiment over time. For the negative sentiment, it peaked around March 2020, when COVID-19 infections started to spread nationally in the United States, and in March 2021. At these 2 time points, positive sentiment rates were at their lowest. In addition, the proportion of New York City tweets made up approximately three-fourths of the total New York tweets, and these 2 displayed similar trajectories in terms of both positive and negative sentiment rates (with *r*=0.97 for both positive and negative sentiment between New York City and New York).

Table 1 shows the model between anti-Asian sentiment and anti-Asian hate crimes in the same month. The crude model 1.0, with a 0-month lag, demonstrates that a 1% point increase in negative sentiment rate toward the Asian community in the same month was associated with a 24% increase (incidence rate ratio [IRR] 1.24; 95% CI 1.07-1.44; *P*=.005) in the number of anti-Asian hate crimes. The significant association persisted but was slightly attenuated after adjusting for unemployment and COVID-19 emergence (ie, after March 2020) in adjusted model 1.1, whereby each percentage point increase in negative sentiment was associated with a 16% increase (IRR 1.16; 95% CI 1.06-1.28; *P*=.008) in expected anti-Asian hate crimes. The positive sentiment toward Asian tweets with a 0-month lag was associated with a 12% decrease (IRR 0.88; 95% CI 0.79-0.97; *P*=.008) in expected anti-Asian hate crimes in the same month in crude model 2.0, and the relationship was no longer significant after adjusting for the unemployment rate and the emergence of COVID-19 pandemic, as shown in model 2.1 (*P*=.11). In addition, there are low to moderate correlations among the independent variables, as shown in Multimedia Appendix 2.

**Table 1:** Results of negative binomial models examining the association between Twitter sentiment rate (negative and positive) and the anti-Asian hate crimes in New York City from 2019 to 2022.

Model^a^ (N=48)			Incidence rate ratio (95% CI)		*P* value
**Crude model 1.0**					
	Negative sentiment (0-month lag)	1.24 (1.07-1.44)		.005	
**Crude model 2.0**					
	Positive sentiment (0-month lag)	0.88 (0.79-0.97)		.008	
**Adjusted model 1.1**					
	Negative sentiment (0-month lag)	1.16 (1.06-1.28)		.002	
**Adjusted model 2.1**					
	Positive sentiment (0-month lag)	0.93 (0.86-1.02)		.11	

^a^The crude models 1.0 and 2.0 regress the number of anti-Asian hate crimes on negative or positive sentiment with a 0-month lag. The adjusted models 1.1 and 2.1 control for the monthly unemployment rate and whether it was during the COVID-19 pandemic (using a 0 value for periods before March 2020 and a value of 1 from March 2020 onward). All models use monthly data and measure sentiment in percentages.

With the assumption that the sentiment on social media may affect hate crimes over time, Multimedia Appendix 3 presents a cross-correlation table between the number of anti-Asian hate crimes per month and sentiment of Twitter data in New York City regarding the Asian community using Pearson *r*. The results suggest a complex relationship where immediate correlations (eg, 0-month lag) are stronger, but there are also potential delayed associations between Twitter sentiment and the number of anti-Asian hate crimes. We further conducted a sensitivity analysis using distributed lag models (presented in Multimedia Appendix 4) adding in a 1- and 2-month lag in addition to a 0-month lag time. For the model with a 0-, 1-, and 2-month lag, a 1% point increase in negative sentiment toward the Asian community with a 0-month lag was associated with a 15% increase (IRR 1.15; 95% CI 1.01-1.31; *P*=.04) in anti-Asian hate crimes. Positive sentiment toward the Asian community on Twitter was associated with an 11% (IRR 0.89; 95% CI 0.81-0.97; *P*=.01) decrease in anti-Asian hate crimes with a 0-month lag. There was no significant association between negative or positive sentiment toward the Asian community and anti-Asian hate crimes when examining 1- to 2-month lag times (all *P*>.05).

## Discussion

### Principal Findings

This study investigates how Twitter sentiment toward the Asian community was associated with anti-Asian hate crimes before and during the COVID-19 pandemic in New York City. We found a positive association between the negative Twitter sentiment rate and the counts of anti-Asian hate crimes in the same month. Positive sentiment toward the Asian community was not associated with hate crimes when adjusted for covariates. Our study highlights the potential of using social media data in predicting and addressing societal issues, including hate crimes. The finding that negative sentiment toward the Asian community was associated with a higher number of anti-Asian hate crimes thereafter may be due to several reasons; it is possible that increased anti-Asian negative sentiment on Twitter could serve as an early indicator of the overall societal climate of racism and discrimination, which were shown in various forms, including hate crimes in the following period. In addition, Twitter can spread and amplify existing prejudices and discriminative views, leading to offline actions such as hate crimes that reflect these attitudes. Supporting this, a study by Nguyen et al [18] demonstrated that residing in a state with a 10% higher negative sentiment in tweets referencing Black individuals was linked to lower odds of individuals endorsing that Black-White disparities in jobs, income, and housing stem from discrimination and higher odds of endorsing that disparities are due to lack of willpower, as well as higher explicit and implicit racial biases. Furthermore, increased negative sentiment on Twitter could represent growing feelings of fear, anger, and vulnerability within Asian communities in response to racism and discrimination, serving as another indicator for the subsequent rise in hate crimes.

The pandemic likely exacerbated anti-Asian hate crimes, potentially due to rhetoric from the media blaming Asian communities for the COVID-19 pandemic, thus fostering an environment that may have facilitated anti-Asian discrimination and violence. March 2020 and March 2021 witnessed peaks in negative sentiment and sharp declines in positive sentiment. March 2020 aligns with the initial COVID-19 outbreak and pandemic declaration by the WHO, and there was a surge of stigmatizing language in the media during this period, including the emergence and use of the phrase “Chinese virus” by public officials and news media [20,33], which likely contributed to the dramatic spike in negative sentiment and drop in positive sentiment as the pandemic emerged. In March 2021, anti-Asian stigma persisted nearly 1 year into the pandemic. In addition, high-profile incidents such as the Atlanta spa shootings in March 2021 coincided with increased awareness of enduring bias and discrimination toward Asian individuals, a sentiment that was reflected in online discussions on Twitter [19].

We are among the first to quantitatively analyze the use of Twitter sentiment data to model anti-Asian hate crimes in the context of the COVID-19 pandemic and to identify the links between online expressions of negative anti-Asian sentiment on social media and offline anti-Asian hate. Although the relationship between social media data and real-world hate crimes has been investigated for some time, it is only recently that social media data have been used in research on anti-Asian hate during the COVID-19 pandemic. Arguing that “racism is a virus,” He et al [34] collected anti-Asian hate and counter speech in tweets and measured the probability of a user becoming hateful (ie, tweeting hate speech for the first time) after being exposed to different percentages of hateful versus counterspeech neighbors in the network. The study found that counterspeech messages discouraged users from adopting hateful attitudes.

Our research highlights how societal crises such as pandemics may potentially exacerbate prejudices, racism, and xenophobia—a phenomenon that is not new in history [35]. Crises like the COVID-19 pandemic may lead to more social cleavages, as evidenced by racist sentiments on social media platforms. This could then contribute to hate crimes targeting minority communities. As an important subject of public health, hate crimes are linked to various psychological and physical harm such as depression, anxiety, stress, and alcohol or drug use [36]. We underscore the urgent need for public health policies that address the compound effects of such experiences. It is important for communities, governments, and social media companies to proactively implement targeted interventions, launch education campaigns, and establish regulations aimed at combating prejudice and discrimination and fostering social cohesion.

This study is not without limitations, but it paves the way for future research directions. First, our research relied upon reported hate crimes, which are often underreported, and Asian survivors are less likely to report hate crimes compared with non-Asian survivors [22]. Therefore, the current count of hate crimes may be an underestimate. Second, there were temporal and geographical limitations concerning granularity. The models evaluated hate crimes and Twitter sentiment at the city level, disregarding finer spatial variations due to the limitations of Twitter data. In addition, the time unit was a month, and subsequent studies could use weekly or daily data, which could provide more units for the modeling. Future research could also benefit from incorporating more granular demographic and socioeconomic data if available. Furthermore, our models using a 0-month lag could not determine causal directionality, as they did not establish whether the increase in anti-Asian Twitter sentiment preceded or followed the rise in anti-Asian hate crimes. In addition, while we used dichotomized time variables to indicate the start of the COVID-19 pandemic, future research might benefit from using other indicators, such as the number of COVID-19 pandemic cases, to further explore their potential influence. Future research could also expand upon our exploratory research and delve deeper into analyses that examine causality.

Twitter users are not representative of the general US population, as younger and higher socioeconomic populations are overrepresented on Twitter [37]. While the sentiment of tweets does not necessarily specify racial prejudice, through this approach, we were able to capture a broad signal of public attitudes, providing insights that may be indicative of a proxy of larger societal trends. It is also important to note the role of social bots on social media. Deceptive bots can manipulate discussions and potentially skew social media study results, a factor that warrants further consideration [38]. However, it can be challenging for the public to differentiate between a bot and a human posting a message. Bots can help create the social context on online social media platforms, which is important to capture. Eliminating bots from the dataset would mean not capturing this exposure. In addition, bots may represent only a small portion of overall social media activity. In our race-related dataset of tweets from 2011 to 2021, we previously investigated the presence of highly frequent users, which can indicate the presence of bots. We found that users tweeting more than 1000 times per year represented less than 1% of the data [39].

Studies have also shown a surge in hate speech following Musk’s acquisition of Twitter in 2022, which could potentially skew the findings of this study [40-42]. This implies that the sentiments expressed on Twitter, especially negative ones, may not be directly comparable before and after the takeover without certain adjustments. Future research should take into account such significant events that could potentially impact social media sentiment when investigating its correlation with real-world crimes. Furthermore, changes in Twitter’s API access and user behavior following Musk’s takeover in 2023 [43-45] pose challenges to the replication of this study and the general use of Twitter data for research purposes. However, our study provides a method framework that can be applied to other social media platforms or data sources. The landscape of social media is dynamic and constantly evolving, and researchers must adapt to these changes. Future research should explore alternative data sources, consider the potential biases introduced by changes in social media platforms, and develop methods to ensure the validity and reliability of social media data for research.

### Public Health Implications

This study underscores the association between negative Twitter sentiment toward the Asian community and anti-Asian hate crimes in New York City, particularly in the context of the COVID-19 pandemic. Most importantly, we demonstrate that social media data can offer new insights for public health in the face of hate crimes. Early detection of spikes in sentiment could help with timely interventions, reducing the negative impacts on the well-being of communities resulting from hate crimes. Overall, it is crucial for governments, social media companies, and communities to address both the immediate health consequences of hate crimes and the broader issue of societal discrimination, and social media data can serve as an effective surveillance tool.

## Appendix

Multimedia Appendix 1 Calculation of precinct-level population proportion.

Multimedia Appendix 2 Correlation table for independent variables (without lag).

Multimedia Appendix 3 Cross-correlation between the number of anti-Asian hate crimes per month and sentiment of Twitter data in New York City regarding the Asian community (using Pearson r). (A) Cross-correlation for the number of anti-Asian hate crimes per month and negative Twitter sentiment at different lag times. (B) Cross-correlation for the number of anti-Asian hate crimes per month and positive Twitter sentiment at different lag times.

Multimedia Appendix 4 Model results for New York City negative and positive sentiment rate using distributed lag regression (n=46 months).

## Abbreviations

API: application programming interface
IRR: incidence rate ratio
WHO: World Health Organization

## Data Availability

The datasets using hate crime data analyzed during this study were provided by the New York City Police Department and are publicly available. The data from X were collected using X’s application programming interface (API) for Academic Research. The API for Academic Research was discontinued in May 2023. X data analyzed during this study cannot be shared because X restricts content distribution.
