# Supplementary material for: From Tweets to Streets: Observational Study on the Association Between Twitter Sentiment and Anti-Asian Hate Crimes in New York City from 2019 to 2022
Source: J Med Internet Res. 2024 Sep 9;26:e53050. doi: 10.2196/53050 (PMC11420573; doi:10.2196/53050)
Supplement: Multimedia Appendix 1 [file jmir_v26i1e53050_app1.docx]

We calculated the precinct-level Asian population proportion using the census block level demographic data from the 2020 Decennial Census [1] and the boundary of NYC Police Precinct [2]. The Asian population and the total population at the precinct level are aggregated using the census block level data, and the proportion was obtained by dividing the Asian population by the total population at the precinct level after aggregation.

Reference:

1. 2020 Decennial Census. US Census Bureau URL: https://data.census.gov/all?q=redistricting [accessed 2024-07-29]

2. NYPD Hate Crimes. NYC Open Data URL: https://data.cityofnewyork.us/Public-Safety/NYPD-Hate-Crimes/bqiq-cu78 [accessed 2024-07-29]
