# Supplementary figures and images for: From Tweets to Streets: Observational Study on the Association Between Twitter Sentiment and Anti-Asian Hate Crimes in New York City from 2019 to 2022
Source: J Med Internet Res. 2024 Sep 9;26:e53050. doi: 10.2196/53050 (PMC11420573; doi:10.2196/53050)

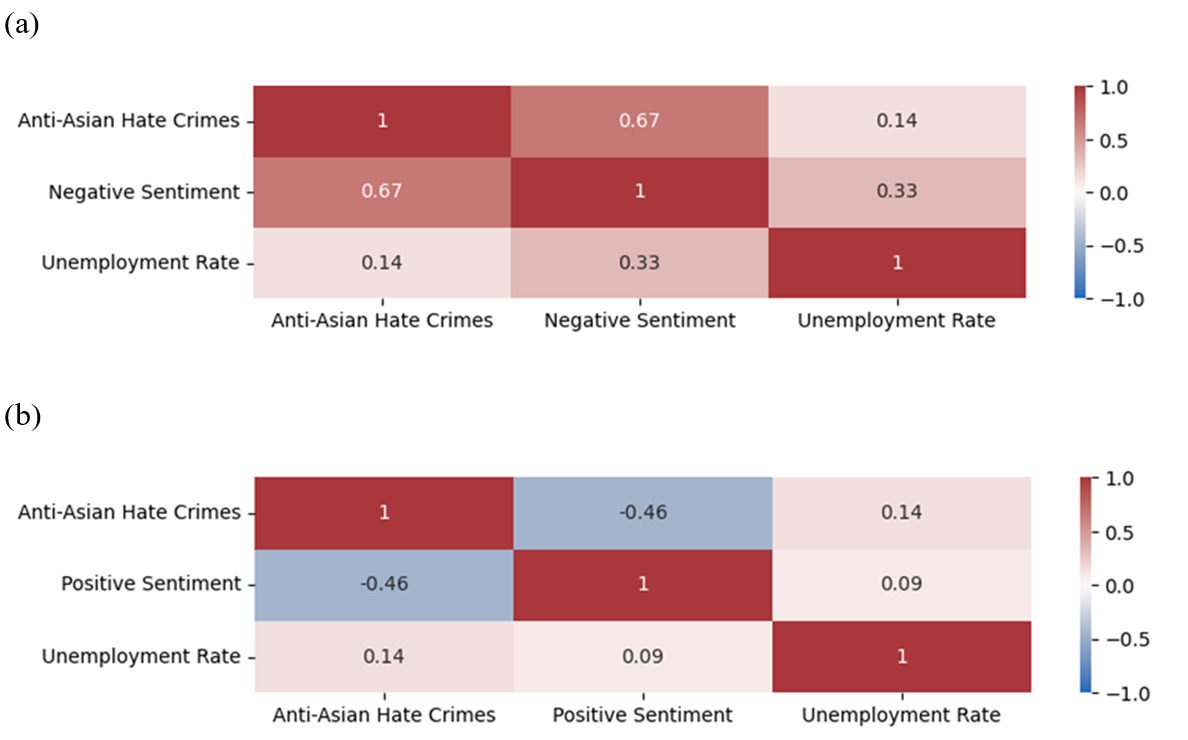

Supplement: Multimedia Appendix 2 [file jmir_v26i1e53050_app2.png]

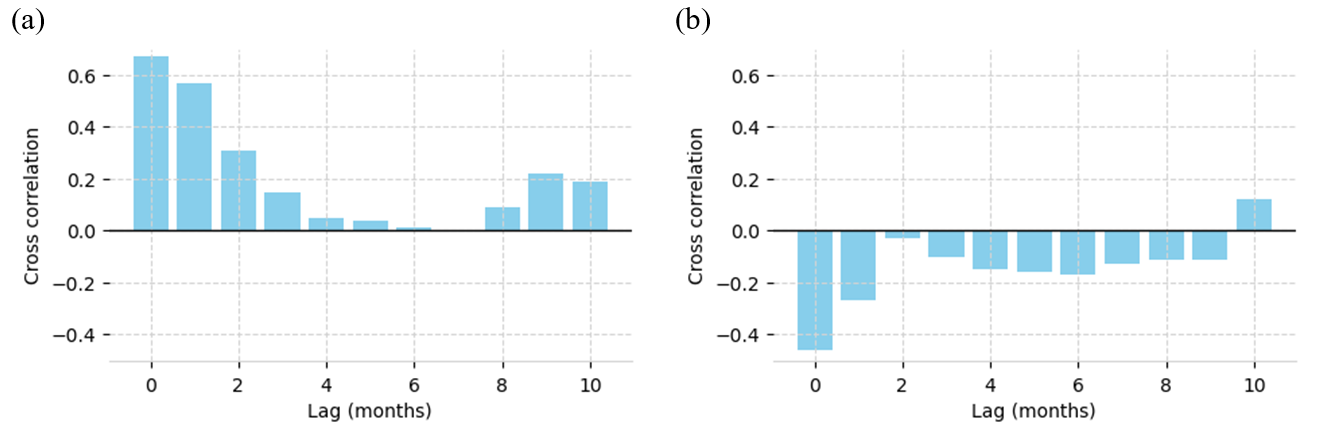

Supplement: Multimedia Appendix 3 [file jmir_v26i1e53050_app3.png]
