# Supplementary material for: From Tweets to Streets: Observational Study on the Association Between Twitter Sentiment and Anti-Asian Hate Crimes in New York City from 2019 to 2022
Source: J Med Internet Res. 2024 Sep 9;26:e53050. doi: 10.2196/53050 (PMC11420573; doi:10.2196/53050)
Supplement: Multimedia Appendix 4 [file jmir_v26i1e53050_app4.docx]

|  | N | Incident Rate Ratio (95% CI) | *P* value |
| --- | --- | --- | --- |
| *Distributed lag model – unadjusted* |  |  |  |
| Negative sentiment (0-month lag) | 46 | 1.15 (1.01, 1.31) | 0.038 |
| Negative sentiment (1-month lag) |  | 1.10 (0.94, 1.29) | 0.231 |
| Negative sentiment (2m lag) |  | 1.05 (0.92, 1.19) | 0.484 |
| *Distributed lag model –unadjusted* |  |  |  |
| Positive sentiment (0-month lag) | 46 | 0.89 (0.81, 0.97) | 0.011 |
| Positive sentiment (1-month lag) |  | 0.89 (0.79, 1.01) | 0.082 |
| Positive sentiment (2-month lag) |  | 0.97 (0.87, 1.09) | 0.658 |

The distributed lag models use 0-month, 1-month and 2-month lags, regressing negative and positive sentiment towards Asians on anti-Asian hate crimes.
